# IL-7 Deficiency Exacerbates Atopic Dermatitis in NC/Nga Mice

**DOI:** 10.3390/ijms24129956

**Published:** 2023-06-09

**Authors:** Hyun Jung Park, Sung Won Lee, Luc Van Kaer, Myeong Sup Lee, Seokmann Hong

**Affiliations:** 1 Department of Integrative Bioscience and Biotechnology, Institute of Anticancer Medicine Development, Sejong University, Seoul 05006, Republic of Korea; 0402parkhj@gmail.com; 2 Department of Biomedical Laboratory Science, College of Health and Biomedical Services, Sangji University, Wonju 26339, Republic of Korea; sungwonlee@sangji.ac.kr; 3 Department of Pathology, Microbiology and Immunology, Vanderbilt University School of Medicine, Nashville, TN 37232, USA; luc.van.kaer@vumc.org; 4 Galux Inc., Gwanak-gu, Seoul 08738, Republic of Korea; myeongsup.lee@gmail.com

**Keywords:** IL-7, atopic dermatitis, NC/Nga mice, CD4^+^ T cells, CD8^+^ T cells, IFN-γ, IL-17

## Abstract

Interleukin-7 (IL-7) plays a vital role in the homeostasis of CD4^+^ and CD8^+^ T cells. Although IL-7 has been implicated in T helper (Th)1- and Th17-mediated autoinflammatory diseases, its role in Th2-type allergic disorders, such as atopic dermatitis (AD), remains unclear. Thus, to elucidate the effects of IL-7 deficiency on AD development, we generated IL-7-deficient AD-prone mice by backcrossing IL-7 knockout (KO) B6 mice onto the NC/Nga (NC) mouse strain, a model for human AD. As expected, IL-7 KO NC mice displayed defective development of conventional CD4^+^ and CD8^+^ T cells compared with wild type (WT) NC mice. However, IL-7 KO NC mice presented with enhanced AD clinical scores, IgE hyperproduction, and increased epidermal thickness compared with WT NC mice. Moreover, IL-7 deficiency decreased Th1, Th17, and IFN-γ-producing CD8^+^ T cells but increased Th2 cells in the spleen of NC mice, indicating that a reduced Th1/Th2 ratio correlates with severity of AD pathogenesis. Furthermore, significantly more basophils and mast cells infiltrated the skin lesions of IL-7 KO NC mice. Taken together, our findings suggest that IL-7 could be a useful therapeutic target for treating Th2-mediated skin inflammations, such as AD.

## 1. Introduction

Atopic dermatitis (AD) is a Th2-mediated chronic inflammatory skin disease characterized by pruritus, xerosis, erythematosus, and edema. Around 5–15% of all children worldwide, including South Korea, suffer from AD. Apart from genetic factors, such as filaggrin gene mutation, environmental factors (i.e., diet, pollutants, and pathogens) can affect the incidence of AD. Skin barrier function is generally impaired in AD, leading to increased epidermal proliferation and infiltration of Th2-type innate immune cells (i.e., mast cells and basophils). Increased serum IgE levels in AD patients correlate with the severity and relapse of AD. Mast cells and basophils are responsible for eliciting Th2 immune responses in AD due to the high affinity of the IgE receptor (FcεR1) [1,2,3,4]. Allergic immune responses in AD are promoted by Th2 cell-derived IL-4 and IL-5, which can be inhibited by cytokines produced from Th1 and regulatory T (Treg) cells. Thus, the development of AD is closely linked to an imbalance in the ratios of Treg/Th2 cells and Th1/Th2 cells and modulating this balance can affect AD treatment and recovery [5,6,7].

Interleukin-7 (IL-7) is a critical cytokine in the development and expansion of T cells. It is mainly secreted by thymic and bone marrow stromal cells and plays a significant role in health maintenance and disease prevention [8]. IL-7 signaling deficiency can result in severe immunodeficiency and, following antigen exposure, contributes to memory T cell development and long-term T cell survival [9]. The IL-7 receptor (IL-7R) is a heterodimeric complex consisting of a unique α-chain and the common γ-chain (γc), which is shared with the receptors for IL-2, IL-4, IL-7, IL-9, IL-15, and IL-21. While IL-2R is expressed on CD4^+^ T cells, CD8^+^ T cells, and Treg cells, IL-7R is mainly expressed on CD4^+^ T cells and CD8^+^ T cells but not Treg cells [10,11]. In addition, IL-7R signaling can impair the differentiation and suppressive function of Foxp3^+^ Treg cells, and an increase of surface IL-7R expression promotes the skewing of CD4^+^ T cell differentiation into Th17 cells [12].

Previous studies have reported that IL-7Rα blockade can mitigate autoimmune diabetes by promoting the proportion of programmed death-1 (PD-1)-expressing effector/memory T cells and Treg cells [13]. In addition, IL-7 signaling is necessary to induce the differentiation of Th1 cells rather than Th17 cells, and serum IL-7 levels correlate inversely with responsiveness to IFN-β therapy in Th1-mediated experimental autoimmune encephalomyelitis (EAE), a mouse model of multiple sclerosis (MS) [14]. Moreover, IL-7 mediates the production of pro-inflammatory cytokines by intra-articular T cells, indicating that IL-7 contributes to increased Th1 responses in patients with rheumatoid arthritis [15].

NC/Nga (NC) mice have been widely employed as a spontaneous murine AD model. When NC mice are maintained under conventional animal housing conditions, they display symptoms similar to AD patients. However, these AD symptoms do not develop under specific pathogen-free (SPF) conditions, suggesting that not only genetic factors but also certain environmental factors (i.e., allergens and bacterial infection) might trigger AD symptoms in NC mice [16].

While most studies on IL-7 have focused on Th1- or Th17-mediated autoinflammatory diseases, its role in Th2-mediated diseases, such as AD, is not fully understood. Therefore, in this study, we investigated the immunoregulatory effects of IL-7 on effector T cells in AD development.

## 2. Results

### 2.1. IL-7 Deficiency Exacerbates AD Pathogenesis in NC/Nga Mice

**Figure 1:**
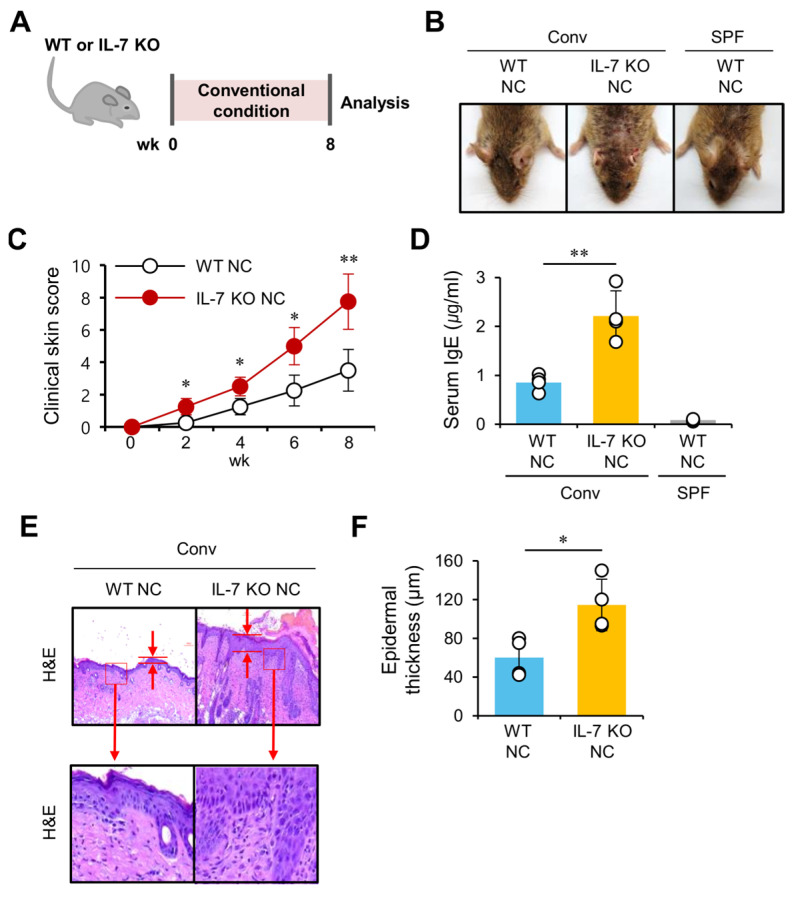
Exacerbation of AD development in IL-7 KO NC mice. (**A**) Experimental outline. WT or IL-7 KO NC mice were allowed to develop AD spontaneously under conventional housing conditions as described in Materials and Methods. WT NC mice housed under specific pathogen-free (SPF) conditions were used as a negative control for AD development. (**B**,**C**) The clinical symptoms were measured every two weeks to monitor the onset of AD. (**D**) Serum IgE levels were measured using ELISA. (**E**,**F**) Skin samples were prepared from WT NC or IL-7 KO NC mice. (**E**) The skin lesions were sectioned and stained with H&E. (**F**) The epidermal thickness was measured in 10 random high-power fields (400×) per sampled lesion using Image J software (version 1.53s, imagej.nih.gov/ij/; accessed on 16 June 2022). The mean values ± SD (*n* = 4 in A–F; per group in the experiment; Student’s *t*-test; * *p* < 0.05, ** *p* < 0.01) are shown. One representative experiment of two experiments is shown.

A previous study demonstrated that IL-7 signaling blockade using anti-IL-7Rα monoclonal antibody (mAb) treatment or endothelial-specific deletion of IL-7Rα attenuates psoriasis-like skin inflammation mediated by infiltration of T cells and DCs into the inflamed lesion [17]. Based on these promising findings, we investigated whether IL-7 plays a role in another skin inflammatory disease, AD. To address this issue, we generated IL-7 knockout (KO) NC mice by backcrossing the mutant IL-7 allele from mice on a B6 genetic background onto the AD-susceptible NC genetic background. Both wild-type (WT) and IL-7 KO NC mice developed skin inflammation spontaneously when raised under conventional housing conditions for eight weeks (Figure 1A). While being maintained under conventional housing conditions, these mice were monitored to measure the clinical skin score, including lichenification, edema, erosion/excoriation, scarring/dryness, and erythema/hemorrhage, every two weeks from six to fourteen weeks of age. While AD severity in WT NC mice increased moderately, IL-7 KO NC mice exhibited a more severe form of AD with increased skin inflammation (Figure 1B). Moreover, IL-7 KO NC mice displayed increased clinical disease scores and serum IgE levels compared with WT NC mice (Figure 1C,D). In addition, we measured the epidermal thickness in hematoxylin and eosin (H&E)-stained skin sections of both WT and IL-7 KO NC mice with AD. The skin lesions of IL-7 KO NC mice displayed a significant increase in epidermal thickness compared with those of WT NC mice (Figure 1E,F). WT NC mice housed under SPF conditions were used as a negative control for AD development. As expected, no clinical AD symptoms were detected in SPF NC mice (Figure 1B,D). Our findings suggest that IL-7 deficiency contributes to the modulation of AD development based on our clinical observations of skin inflammation and serum IgE levels.

### 2.2. IL-7 Deficiency Induces Impaired Development of CD4^+^ and CD8^+^ T Cells in NC Mice with AD

**Figure 2:**
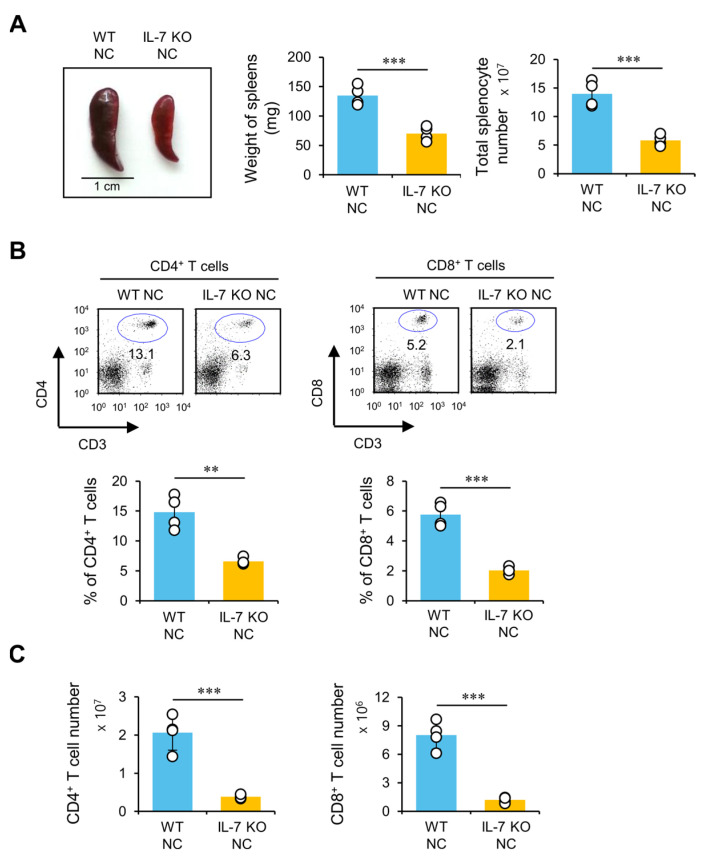
Impaired T cell development in IL-7 KO NC mice with AD. (**A**–**C**) WT NC and IL-7 KO NC mice were transferred to the conventional housing conditions at six weeks of age to develop AD spontaneously. Splenocytes were prepared from these mice at 14 weeks of age. (**A**) (Left), A representative picture of the spleens from AD-induced WT NC and IL-7 KO NC mice. (Middle) and (Right), Spleen weights and splenocyte numbers of these mice. (**B**,**C**) The percentages and the absolute total cell numbers of both CD4^+^ and CD8^+^ T cells were analyzed via flow cytometry. The mean values ± SD (*n* = 4 in A-C; per group in the experiment; Student’s *t*-test; ** *p* < 0.01, *** *p* < 0.001) are shown. One representative experiment of two experiments is shown.

As it has been reported that IL-7 signaling can enhance the survival of T cells by up-regulating anti-apoptotic Bcl-2 and down-regulating pro-apoptotic Bim [18], we examined whether IL-7 deficiency affects T cell profiles (phenotypes) in NC mice. For this purpose, total T cells were isolated from NC mice with AD and assessed for CD4 and CD8 expression using flow cytometry. We found that IL-7 KO NC mice displayed smaller spleen size and decreased frequency of splenocytes (Figure 2A) when they developed AD. Furthermore, we found that the number and frequency of both CD4^+^ T cells and CD8^+^ T cells were significantly lower in IL-7 KO NC mice than in WT NC mice during AD development (Figure 2B,C). To evaluate the effect of AD development on impaired T cell development mediated by IL-7 deficiency, we compared the extent of T cell development in WT NC and IL-7 KO NC housed under SPF conditions. We found that IL-7 deficiency-mediated impairment of T cell development was not affected by AD development (Figure S1). Interestingly, however, both WT NC and IL-7 KO NC mice with AD displayed reduced CD4^+^ and CD8^+^ T cell numbers compared with their respective controls without AD. Consistent with previous studies, our results indicate that IL-7 is essential in maintaining T cells in NC mice under both SPF and conventional housing conditions. Thus, it appears that exacerbated AD pathogenesis in IL-7 KO NC mice is not attributed to a simple quantitative reduction of T cells but rather to qualitative changes of T cell pools.

### 2.3. Worsened AD Pathogenesis in IL-7 KO NC Mice Is Associated with Th2-Baised Immune Responses and Down-Regulation of IFN-γ and IL-17 Expression

**Figure 3:**
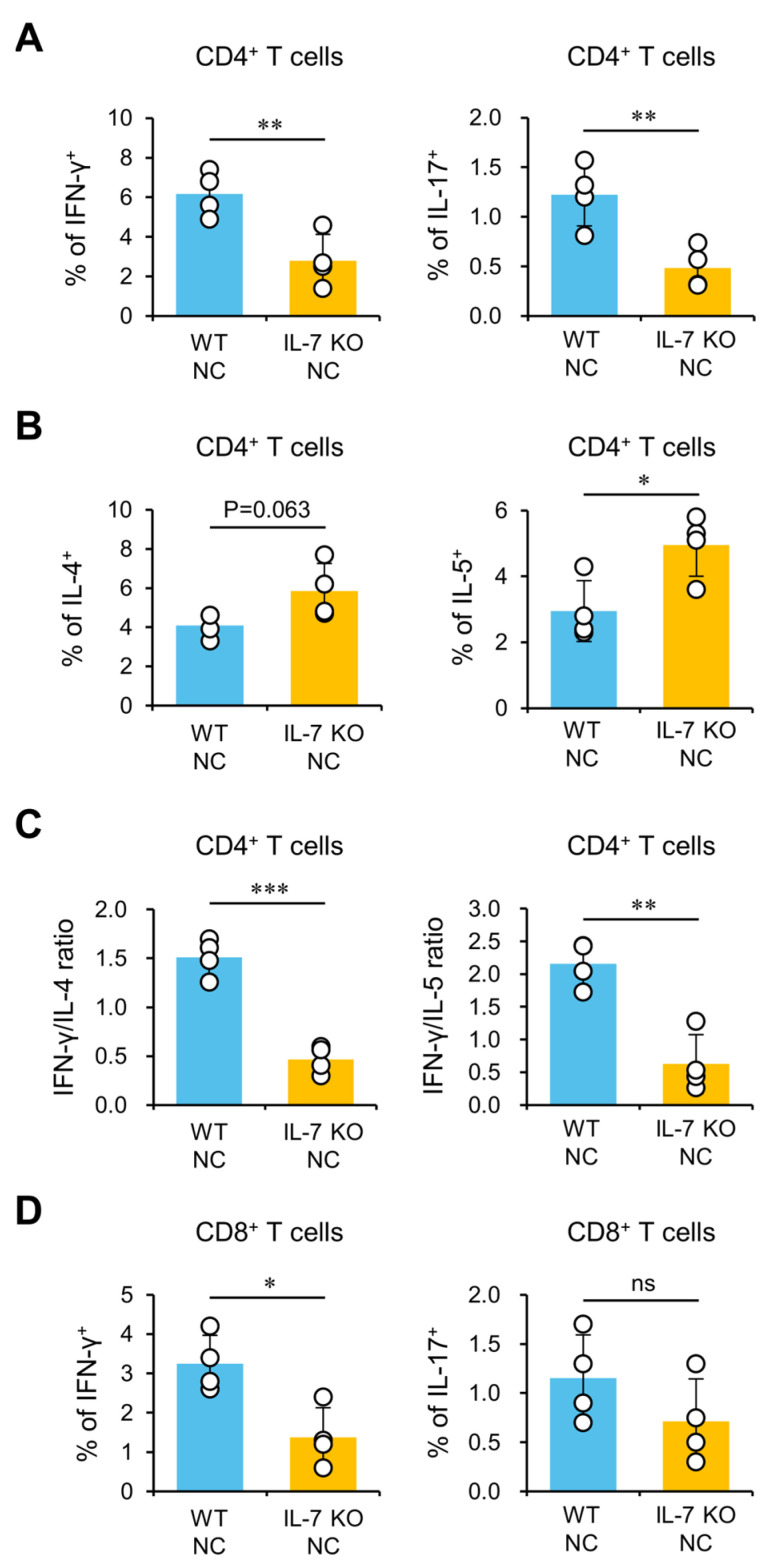
Down-regulation of IFN-γ and IL-17 expression may be linked to up-regulation of Th2-biased immune responses in IL-7 KO NC mice. (**A**–**D**) WT NC and IL-7 KO NC mice were transferred to the conventional housing conditions at six weeks of age to develop AD spontaneously. Splenocytes were prepared from these mice at 14 weeks of age. (**A**) IFN-γ- and IL-17-producing subpopulations among splenic CD4^+^ T cells from each group were determined using flow cytometry. (**B**) IL-4- and IL-5-producing subpopulations among splenic CD4^+^ T cells were evaluated using flow cytometry. (**C**) The ratio of the IL-4- or IL-5-producing population to the IFN-γ-producing population in splenic CD4^+^ T cells was measured using flow cytometric analysis. (**D**) IFN-γ- and IL-17-producing subpopulations among splenic CD8^+^ T cells from each group were determined using flow cytometric analysis. The mean values ± SD (*n* = 4 in A–D; per group in the experiment; Student’s *t*-test; * *p* < 0.05, ** *p* < 0.01, *** *p* < 0.001) are shown. One representative experiment of two experiments is shown. ns, not significant.

As previous studies have shown that elevated Th2 immune responses are responsible for AD pathogenesis [5], we investigated whether IL-7 deficiency impacts Th2 cell differentiation during AD development. To explore this possibility, we performed flow cytometric analyses to determine Th1/Th2 cytokine profiles of CD4^+^ T cells from IL-7 KO NC mice with fully developed AD. We found that IL-7 KO NC mice with AD exhibit reduced production of IFN-γ and IL-17 cytokines in CD4^+^ T cells compared to WT NC mice, suggesting that Th1 and Th17 cell differentiation are negatively affected in IL-7 KO NC mice during AD development (Figure 3A). Next, we also measured Th2 cytokines (IL-4 and IL-5) in CD4^+^ T cells to examine whether exacerbated AD in IL-7 KO NC mice is related to changes in Th2 cells. We found that IL-7 KO NC mice display elevated IL-4 and IL-5 expression in CD4^+^ T cells following AD induction compared to WT NC mice (Figure 3B). IL-7 KO NC mice displayed a reduced Th1/Th2 ratio, indicating that Th2-biased immune responses dominate in these mice (Figure 3C). Interestingly, we also found decreased IFN-γ-producing but not IL-17-producing CD8^+^ T cells in the IL-7 KO NC mice, similar to the observed reduction in Th1 cells (Figure 3D). In addition, to evaluate the effect of AD susceptibility (but not active AD) on T cell cytokine production, we compared cytokine production of CD4^+^ and CD8^+^ T cells in WT NC and IL-7 KO NC mice housed under SPF conditions. We found that IL-7 KO NC without AD display a reduction in IFN-γ and IL-17 production by CD4^+^ T cells. However, unlike WT NC and IL-7 KO NC mice with AD, the control NC mice housed under SPF conditions did not show any significant changes in IL-4 and IL-5 expression by CD4^+^ T cells, which indicates that exacerbation of AD might be attributed to environmental factors, such as microbial infections (Figure S2). These results indicate that IL-7 deficiency up-regulates pathogenic Th2 immune responses but down-regulates Th1 and CD8^+^ T cells in NC mice during AD development.

### 2.4. The Effects of IL-7 Deficiency on AD Severity Are Associated with Expanded Basophils and Mast Cells in the Skin

**Figure 4:**
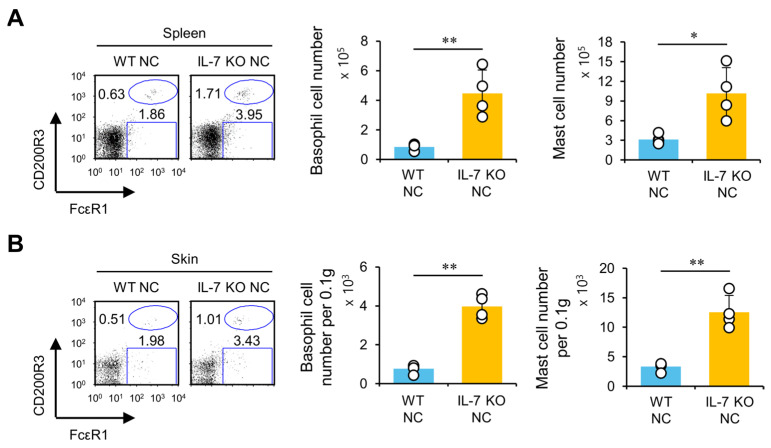
IL-7 deficiency is associated with expanded basophils and mast cells in NC mice with AD. (**A**) Spleens were prepared from mice as shown in Figure 2. The frequency and total absolute cell number of splenic basophils (FcεR1^+^CD200R3^+^CD3^−^CD19^−^) and mast cells (FcεR1^+^CD200R3^−^CD3^−^CD19^−^) were determined using flow cytometry. (**B**) Mononuclear cells (MNCs) in the skin were isolated from mice as shown in Figure 1. The absolute cell numbers of basophils (CD45^+^FcεR1^+^CD200R3^+^CD3^−^CD19^−^) and mast cells (CD45^+^FcεR1^+^CD200R3^−^CD3^−^CD19^−^) were determined in the skin using flow cytometry. The mean values ± SD (*n* = 4 in A,B; per group in the experiment; Student’s *t*-test; * *p* < 0.05, ** *p* < 0.01) are shown. One representative experiment of two experiments is shown.

Basophils and mast cells can contribute to allergic immune responses through IgE-mediated FcεR1 stimulation. Moreover, basophils can enhance Th2 polarization by quickly secreting IL-4. Thus, we investigated whether these innate immune cells correlate with exacerbated AD pathogenesis in IL-7 KO NC mice. As expected, splenic basophils and mast cells were significantly increased in number in IL-7 KO NC mice than in WT NC mice during AD development (Figure 4A). Additionally, IL-7 KO NC mice had a more significant infiltration of basophils and mast cells in skin lesions (Figure 4B). These findings indicate that IL-7 deficiency promotes Th2-mediated immune responses and consequently expands allergic effectors, such as basophils and mast cells, ultimately exacerbating spontaneous AD development in IL-7 KO NC mice.

### 2.5. Human Gene Expression Data Suggest a Strong Association between IL-7 Gene Expression and IFN-γ and IL-17 Gene Expression

**Figure 5:**
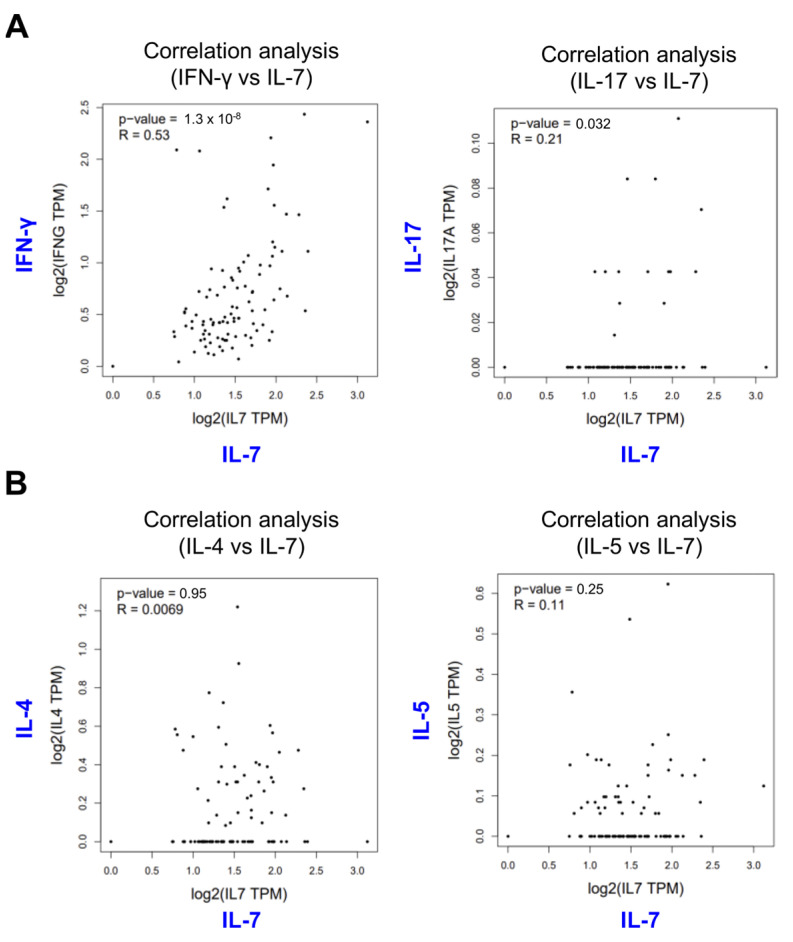
Correlation analyses of *IL-7* and *IFNG* or *IL-17A* gene expression in human spleen. (**A**,**B**) Pearson correlation analysis of *IL-7* and Th-related cytokine genes (*IFNG*, *IL-17A*, *IL-4*, and *IL-5*) was conducted using the human splenic data from the GEPIA (http://gepia.cancer-pku.cn/index.html, accessed on 9 August 2022) tool (TPM; transcripts per million reads).

As our results strongly suggest that IL-7 plays a critical role in determining the effector functions of CD4 and CD8 T cells, we next asked whether IL-7 gene expression might be linked to Th1/Th17 (IFN-γ and IL-17) and Th2 (IL-4 and IL-5) cytokine expression profiles in humans. To examine a possible correlation between IL-7 gene expression and T helper cytokine gene expression (*IFNG*, *IL-17*, *IL-4*, and *IL-5*), we took advantage of the data from the GEPIA (Gene Expression Profiling Interactive Analysis) website containing expression data for human spleen. Our analysis revealed that *IL-7* expression considerably correlated with the expression of *IFNG* (R  =  0.53, *p*  =  1.3 × 10^−8^) and *IL-17A* (R  =  0.21, *p*  =  0.032), but not with *IL-4* and *IL-5* (Figure 5A,B). These results support the notion that IL-7 regulates T helper cell differentiation, ultimately contributing to the outcome of AD pathogenesis.

## 3. Discussion

In this study, we investigated the role of IL-7 on AD development by employing IL-7 KO NC mice. NC mice show an impaired immune response against bacterial skin infections, such as *Staphylococcus aureus* (the leading cause of AD in these mice) [19]. Protective immune responses against invasive *S. aureus* infection require IFN-γ and IL-17 [20,21], which act in synergy to safeguard the oral mucosal layer against *S. aureus* [22]. Our results indicate that the reduction of IFN-γ- and IL-17-producing CD4^+^ T cells in IL-7 KO NC mice may contribute to the breakdown of the skin’s immune defense system against *S. aureus* colonization. Thus, it would be interesting to investigate whether the severe skin inflammation found in IL-7 KO NC mice is attributed to *S. aureus*-dominant colonization in the skin. 

Furthermore, as splenic CD4^+^ T cells from IL-7 KO mice are defective in producing IFN-γ and IL-17 rather than Th2-type cytokines (e.g., IL-4 or IL-5), the decrease of IFN-γ and IL-17 production may trigger the initiation of Th2 immune responses, consequently leading to accelerated AD development in IL-7 KO mice (Figure S2). Moreover, consistent with our results, GEPIA analysis of human spleen showed IL-7 production significantly correlates with IFN-γ or IL-17 but not with IL-4 or IL-5 (Figure 4). These findings suggest that a systemic deficiency of IL-7 down-regulates IFN-γ and IL-17 production by CD4^+^ T cells, likely providing a Th2-conducive environment that might be favorable for AD-exacerbating invasive *S. aureus* infection. 

Systemic oral immunosuppressive drugs are commonly used to treat AD, but more than 70% of AD patients receiving these therapies exhibit lymphopenia, a quantitative decrease in lymphocyte cell numbers (≤1 × 10^3^ cells/μL) [23]. Recent findings also demonstrated that patients with atopic eczema (AE) have lower lymphocyte counts than individuals without AE, suggesting that a decrease in lymphocyte cell numbers significantly correlates with more severe AD [24]. It has been reported that IL-7 therapy can increase functional T cells and reverse profound lymphopenia [25,26]. Moreover, lymphopenia in AD patients shows a tendency for preferential depletion of IFN-γ-producing T cells (both CD4^+^ and CD8^+^ T cells), which might result in Th2-deviated immune responses [27]. Based on the capacity of IFN-γ to negatively regulate both the activation and survival of AD-initiating effector cells (e.g., mast cells and basophils) [28,29], the IL-7 deficiency-mediated reduction in IFN-γ-producing T cells might be responsible for mast cell and basophil activation, which consequently could enhance AD development. As our results indicate that AD development in IL-7 KO NC mice is accompanied by a decreased number of CD4^+^ and CD8^+^ T cells, it would be worthwhile to investigate whether IL-7 is related to the onset of lymphopenia in AD patients.

Intriguingly, a recent study reported that IL-7 is critical for maintaining invariant natural killer T (iNKT) cells, a subset of glycolipid-reactive T cells with innate-like functions, in peripheral tissues, including lymph nodes, spleen, liver, and lung [30]. Previous studies have revealed that iNKT cells are impaired in NC mice due to the absence of the TCR Vβ8 gene [31]. Recently, our studies have shown that Vα14 TCR transgenic (Vα14^Tg^) NC mice overexpressing Vα14 iNKT cells exhibit significantly reduced skin inflammation upon spontaneous AD development compared to WT NC mice [3,32,33]. Furthermore, skin iNKT cells from Vα14^Tg^ NC mice exhibit a Th1-like phenotype with high IFN-γ and IL-2 during AD development [32]. Thus, it might be interesting to examine whether IL-7 deficiency can trigger the protective effects of Vα14^Tg^ iNKT cells on AD development in NC mice.

Our findings indicate that IL-7 KO NC mice develop worsened skin inflammation and a faster disease progression. We propose that the deficiency of IL-7 expression contributes to the development of AD through a series of steps. Firstly, a decrease in IFN-γ- and IL-17-producing CD4^+^ T cells occurs due to reduced IL-7 expression, possibly resulting in uncontrolled *S. aureus* infection. Secondly, IL-4-producing basophils and mast cells expand, which promotes the differentiation of naive CD4^+^ T cells into Th2 cells. Finally, impaired IFN-γ production induced by IL-7 deficiency causes a loss of negative regulation of IL-4 activity, leading to uncontrollable AD development. Consequently, our research demonstrates that IL-7 has the potential to be used as a therapeutic approach for managing inflammatory skin diseases, such as AD. Thus, IL-7 immunotherapy might be an option to substitute steroid therapy, which is being widely used despite its adverse effects. 

## 4. Materials and Methods

### 4.1. Study Design

This study was designed to investigate the effect of IL-7 on the development of AD in IL-7 KO NC mice. To address this issue, AD was allowed to develop spontaneously in IL-7 KO mice and WT controls. Skin leukocytes and splenocytes were then isolated and analyzed using flow cytometry, while sera were harvested and analyzed using ELISA. This study received approval from the Sejong University Institutional Review Board before the experiments were conducted (SJ-20181101E2).

### 4.2. Mice

WT NC mice were purchased from Jung Ang Lab Animal Inc. (Seoul, Republic of Korea). IL-7 KO B6 mice [34] were backcrossed to NC mice for more than ten generations. These mice were maintained at the Sejong University and were used for experiments at 6–12 weeks of age. The mice were maintained on a 12 h light/12 h dark cycle in a temperature-controlled barrier facility with free access to food and water. They were fed a γ-irradiated sterile diet and provided with autoclaved tap water. To monitor spontaneous AD development, mice were transferred to a conventional animal facility at six weeks of age. Age- and sex-matched mice were used for all experiments. The animal experiments were approved by the Institutional Animal Care and Use Committee of Sejong University (SJ-20181101E2).

### 4.3. Flow Cytometry

The following mAbs were obtained from BD Biosciences (San Jose, CA, USA): Phycoerythrin (PE)-, PE-Cy7- or allophycocyanin (APC)-conjugated anti-CD3ε (clone 145-2C11); PE-Cy7- or APC-conjugated anti-CD4 (clone RM4-5); PE-Cy7- or APC-conjugated anti-CD8α (clone 53-6.7); PE-Cy7- or APC-conjugated anti-CD45 (clone 30-F11), PE-conjugated immunoglobulin G (IgG) (isotype control) (clone R3-34). In addition, the following mAbs from Thermo Fisher Scientific (Waltham, MA, USA) were used: APC-conjugated anti-CD200R3 (clone Ba13); PE-Cy7-conjugated anti-CD19 (clone ID3); PE-conjugated anti-FcεR1 (clone MAR-1); PE-conjugated anti-IFN-γ (clone XMG1.2); PE-conjugated anti-IL-4 (clone BVD6-24G2); PE-conjugated anti-IL-5 (clone TRFK5); PE-conjugated anti-IL-17 (clone eBio17B7). To perform surface staining, cells were harvested and washed twice with cold 0.5% BSA-containing PBS (FACS buffer). To block the Fc receptor, the cells were incubated with anti-CD16/CD32 mAbs on ice for 10 min and subsequently stained with fluorescently labeled mAbs. Flow cytometric data were acquired using a FACSCalibur flow cytometer (Becton Dickson, San Jose, CA, USA) and analyzed using FlowJo software (version 8.7; Tree Star, Ashland, OR, USA) [35].

### 4.4. Flow Cytometry

For intracellular staining, splenocytes were incubated with brefeldin A, an intracellular protein transport inhibitor (10 μg/mL), in RPMI medium (Gibco BRL, Gaithersburg, MD, USA) for 2 h at 37 °C. The cells were stained for cell surface markers, fixed with 1% paraformaldehyde, washed once with cold FACS buffer, and permeabilized with 0.5% saponin. The permeabilized cells were then stained for an additional 30 min at room temperature with the indicated mAbs (PE-conjugated anti-IFN-γ, anti-IL-4, anti-IL-5, anti-IL-17, or PE-conjugated isotype control rat IgG) [36]. More than 5000 cells per sample were acquired using a FACSCalibur flow cytometer, and the data were analyzed using the FlowJo software package (version 8.7; Tree Star, Ashland, OR, USA).

### 4.5. ELISA

Serum IgE levels were measured with a sandwich ELISA (clone R35-72 for capturing IgE and R35-118 for detecting IgE; BD PharMingen, San Jose, CA, USA). In addition, the optical density was measured at 450 nm with an immunoreader (Bio-Tek ELX-800, Winooski, VT, USA).

### 4.6. Preparation of Skin Cell Suspensions

The skin was dissected and dermal fat was removed with scissors. The tissue was cut into small pieces with a scalpel and digested with 2.5 mg/mL collagenase type IV (Sigma, St. Louis, MO, USA) and 1 mg/mL DNase I (Promega, Madison, WI, USA) for 4 h at 37 °C. At the end of the incubation, the digested tissue was dissociated into single-cell suspensions using gentleMACS Dissociator (Miltenyi, Bergisch Gladbach, Germany) in combination with C Tubes. Single-cell suspensions were smashed through a 70 μm nylon cell strainer (BD Falcon, Franklin Lakes, NJ, USA) and collected in a 50 mL Falcon tube. The cells were washed once with PBS plus 10 % FBS (1400 rpm, 10 min, 4 °C) and subsequently separated with 37%/70% Percoll (GE Healthcare, Chicago, IL, USA) gradients. Mononuclear cells were collected from the layer below 37% and above 70% gradient. After washing with PBS, the total mononuclear cell number was determined using a hemacytometer with 0.4% trypan blue (Welgene, Gyeongsan-si, Republic of Korea) before antibody staining.

### 4.7. Analysis of Skin Sections

The dorsal skin was fixed in 4% paraformaldehyde, embedded in paraffin, and cut into six μm sections using a microtome (RM 2235, Leica, Wetzlar, Germany). The sections were then stained with hematoxylin and eosin (H&E) to analyze histological changes. The epidermal thickness was measured in ten high-power fields (400×) per each section using Image J software (version 1.53s, imagej.nih.gov/ij/; accessed on 16 June 2022) (National Institutes of Health, Bethesda, MD, USA).

### 4.8. Scoring the Severity of Skin Lesions

Skin lesions were scored at the indicated time points. The scoring was based on the severity of lichenification, edema, erosion/excoriation, scarring/dryness, and erythema/hemorrhage. The total clinical skin severity score was defined as the sum of the five signs (none = 0; mild = 1; moderate = 2; and severe = 3).

### 4.9. Data Collection in the GEPIA (Gene Expression Profiling Interactive Analysis)

Correlation analysis between *IL-7* and Th-related cytokines (*IFNG*, *IL-17A*, *IL-4*, and *IL-5*) was conducted using RNA-seq data of healthy human spleen from a bioinformatics GEPIA database [37]. Data credit: GEPIA. Data summary images were obtained from http://gepia.cancer-pku.cn/index.html (accessed on 9 August 2022).

### 4.10. Statistical Analysis

Statistical significance was determined using Excel (Microsoft, Redmond, WA, USA). Student’s *t*-test was performed for the comparison of two groups (* *p* < 0.05, ** *p* < 0.01, and *** *p* < 0.001 were considered significant in the Student’s *t*-test). Two-way ANOVA analysis was carried out using the VassarStats (http://vassarstats.net/anova2u.html). # *p* < 0.05, ## *p* < 0.01, and ### *p* < 0.001 were considered to be significant in the two-way ANOVA.

## Data Availability

The data will be available from the corresponding author upon reasonable request.

## Supplementary Materials

The following supporting information can be downloaded at: https://www.mdpi.com/article/10.3390/ijms24129956/s1.

## References

[1] Otsuka A., Kabashima K. (2015). Mast cells and basophils in cutaneous immune responses. Allergy.

[2] Lee S.W., Park H.J., Jeon J., Park Y.H., Kim T.C., Jeon S.H., Seong R.H., Van Kaer L., Hong S. (2021). Ubiquitous Overexpression of Chromatin Remodeling Factor SRG3 Exacerbates Atopic Dermatitis in NC/Nga Mice by Enhancing Th2 Immune Responses. Int. J. Mol. Sci..

[3] Park H.J., Kim T.C., Park Y.H., Lee S.W., Jeon J., Park S.H., Van Kaer L., Hong S. (2021). Repeated alpha-GalCer Administration Induces a Type 2 Cytokine-Biased iNKT Cell Response and Exacerbates Atopic Skin Inflammation in Valpha14(Tg) NC/Nga Mice. Biomedicines.

[4] Hadi H.A., Tarmizi A.I., Khalid K.A., Gajdacs M., Aslam A., Jamshed S. (2021). The Epidemiology and Global Burden of Atopic Dermatitis: A Narrative Review. Life.

[5] Park H.J., Lee S.W., Hong S. (2018). Regulation of Allergic Immune Responses by Microbial Metabolites. Immune Netw..

[6] Lee S.W., Park H.J., Park S.H., Hong S. (2013). Oral administration of poly-gamma-glutamic acid prevents the development of atopic dermatitis in NC/Nga mice. Exp. Dermatol..

[7] Weidinger S., Beck L.A., Bieber T., Kabashima K., Irvine A.D. (2018). Atopic dermatitis. Nat. Rev. Dis. Primers.

[8] Schluns K.S., Kieper W.C., Jameson S.C., Lefrancois L. (2000). Interleukin-7 mediates the homeostasis of naive and memory CD8 T cells in vivo. Nat. Immunol..

[9] Kondrack R.M., Harbertson J., Tan J.T., McBreen M.E., Surh C.D., Bradley L.M. (2003). Interleukin 7 regulates the survival and generation of memory CD4 cells. J. Exp. Med..

[10] Lin J.X., Leonard W.J. (2018). The Common Cytokine Receptor gamma Chain Family of Cytokines. Cold Spring Harb. Perspect. Biol..

[11] Park H.J., Lee S.W., Park Y.H., Kim T.C., Van Kaer L., Hong S. (2022). CD1d-independent NK1.1(+) Treg cells are IL2-inducible Foxp3(+) T cells co-expressing immunosuppressive and cytotoxic molecules. Front. Immunol..

[12] Waickman A.T., Keller H.R., Kim T.H., Luckey M.A., Tai X., Hong C., Molina-Paris C., Walsh S.T.R., Park J.H. (2020). The Cytokine Receptor IL-7Ralpha Impairs IL-2 Receptor Signaling and Constrains the In Vitro Differentiation of Foxp3(+) Treg Cells. iScience.

[13] Penaranda C., Kuswanto W., Hofmann J., Kenefeck R., Narendran P., Walker L.S., Bluestone J.A., Abbas A.K., Dooms H. (2012). IL-7 receptor blockade reverses autoimmune diabetes by promoting inhibition of effector/memory T cells. Proc. Natl. Acad. Sci. USA.

[14] Lee L.F., Axtell R., Tu G.H., Logronio K., Dilley J., Yu J., Rickert M., Han B., Evering W., Walker M.G. (2011). IL-7 promotes T(H)1 development and serum IL-7 predicts clinical response to interferon-beta in multiple sclerosis. Sci. Transl. Med..

[15] van Roon J.A., Glaudemans K.A., Bijlsma J.W., Lafeber F.P. (2003). Interleukin 7 stimulates tumour necrosis factor alpha and Th1 cytokine production in joints of patients with rheumatoid arthritis. Ann. Rheum. Dis..

[16] Suto H., Matsuda H., Mitsuishi K., Hira K., Uchida T., Unno T., Ogawa H., Ra C. (1999). NC/Nga mice: A mouse model for atopic dermatitis. Int. Arch. Allergy Immunol..

[17] Vranova M., Friess M.C., Haghayegh Jahromi N., Collado-Diaz V., Vallone A., Hagedorn O., Jadhav M., Willrodt A.H., Polomska A., Leroux J.C. (2019). Opposing roles of endothelial and leukocyte-expressed IL-7Ralpha in the regulation of psoriasis-like skin inflammation. Sci. Rep..

[18] Chetoui N., Boisvert M., Gendron S., Aoudjit F. (2010). Interleukin-7 promotes the survival of human CD4+ effector/memory T cells by up-regulating Bcl-2 proteins and activating the JAK/STAT signalling pathway. Immunology.

[19] Hashimoto Y., Kaneda Y., Akashi T., Arai I., Nakaike S. (2004). Persistence of Staphylococcus aureus colonization on the skin of NC/Nga mice. J. Dermatol. Sci..

[20] Brown A.F., Murphy A.G., Lalor S.J., Leech J.M., O’Keeffe K.M., Mac Aogain M., O’Halloran D.P., Lacey K.A., Tavakol M., Hearnden C.H. (2015). Memory Th1 Cells Are Protective in Invasive Staphylococcus aureus Infection. PLoS Pathog..

[21] Cho J.S., Pietras E.M., Garcia N.C., Ramos R.I., Farzam D.M., Monroe H.R., Magorien J.E., Blauvelt A., Kolls J.K., Cheung A.L. (2010). IL-17 is essential for host defense against cutaneous Staphylococcus aureus infection in mice. J. Clin. Investig..

[22] Barin J.G., Talor M.V., Schaub J.A., Diny N.L., Hou X., Hoyer M., Archer N.K., Gebremariam E.S., Davis M.F., Miller L.S. (2016). Collaborative Interferon-gamma and Interleukin-17 Signaling Protects the Oral Mucosa from Staphylococcus aureus. Am. J. Pathol..

[23] Bakker D.S., Garritsen F.M., Leavis H.L., van der Schaft J., Bruijnzeel-Koomen C., van den Broek M.P.H., de Bruin-Weller M.S. (2018). Lymphopenia in atopic dermatitis patients treated with oral immunosuppressive drugs. J. Dermatol. Treat..

[24] Hollestein L.M., Ye M.Y.F., Ang K.L., Forbes H., Mansfield K.E., Abuabara K., Smeeth L., Langan S.M. (2023). The association between atopic eczema and lymphopenia: Results from a UK cohort study with replication in US survey data. J. Eur. Acad. Dermatol. Venereol..

[25] Mazer M.B., Turnbull I.R., Miles S., Blood T.M., Sadler B., Hess A., Botney M.D., Martin R.S., Bosanquet J.P., Striker D.A. (2021). Interleukin-7 Reverses Lymphopenia and Improves T-Cell Function in Coronavirus Disease 2019 Patient with Inborn Error of Toll-Like Receptor 3: A Case Report. Crit. Care Explor..

[26] Sheikh V., Porter B.O., DerSimonian R., Kovacs S.B., Thompson W.L., Perez-Diez A., Freeman A.F., Roby G., Mican J., Pau A. (2016). Administration of interleukin-7 increases CD4 T cells in idiopathic CD4 lymphocytopenia. Blood.

[27] Lonati A., Licenziati S., Canaris A.D., Fiorentini S., Pasolini G., Marcelli M., Seidenari S., Caruso A., De Panfilis G. (1999). Reduced production of both Th1 and Tc1 lymphocyte subsets in atopic dermatitis (AD). Clin. Exp. Immunol..

[28] Park H.J., Lee S.W., Park S.H., Hong S. (2016). iNKT Cells Are Responsible for the Apoptotic Reduction of Basophils That Mediate Th2 Immune Responses Elicited by Papain in Mice Following gammaPGA Stimulation. PLoS ONE.

[29] Mann-Chandler M.N., Kashyap M., Wright H.V., Norozian F., Barnstein B.O., Gingras S., Parganas E., Ryan J.J. (2005). IFN-gamma induces apoptosis in developing mast cells. J. Immunol..

[30] Park J.Y., Won H.Y., DiPalma D.T., Kim H.K., Kim T.H., Li C., Sato N., Hong C., Abraham N., Gress R.E. (2022). In vivo availability of the cytokine IL-7 constrains the survival and homeostasis of peripheral iNKT cells. Cell Rep..

[31] Habu Y., Shinomiya N., Kinoshita M., Matsumoto A., Kawabata T., Seki S. (2007). Mice deficient in Vbeta8+NKT cells are resistant to experimental hepatitis but are partially susceptible to generalised Shwartzman reaction. Clin. Exp. Med..

[32] Park H.J., Lee S.W., Park S.H., Van Kaer L., Hong S. (2021). Selective Expansion of Double-Negative iNKT Cells Inhibits the Development of Atopic Dermatitis in Valpha14 TCR Transgenic NC/Nga Mice by Increasing Memory-Type CD8(+) T and Regulatory CD4(+) T Cells. J. Investig. Dermatol..

[33] Lee S.W., Park H.J., Van Kaer L., Hong S. (2022). Roles and therapeutic potential of CD1d-Restricted NKT cells in inflammatory skin diseases. Front. Immunol..

[34] Miller C.N., Hartigan-O’Connor D.J., Lee M.S., Laidlaw G., Cornelissen I.P., Matloubian M., Coughlin S.R., McDonald D.M., McCune J.M. (2013). IL-7 production in murine lymphatic endothelial cells and induction in the setting of peripheral lymphopenia. Int. Immunol..

[35] Park H.J., Lee S.W., Song J.G., Van Kaer L., Cheon J.H., Lim S.J., Han H.K., Hong S. (2022). Aminoclay Nanoparticles Induce Anti-Inflammatory Dendritic Cells to Attenuate LPS-Elicited Pro-Inflammatory Immune Responses. Molecules.

[36] Lee S.W., Oh S.Y., Park H.J., Kim T.C., Park Y.H., Van Kaer L., Hong S. (2022). Phosphorothioate-linked guanine/cytosine-based stem-loop oligonucleotides induce the extracellular release of mitochondrial DNA from peritoneal B1a cells. Int. J. Biol. Macromol..

[37] Tang Z., Li C., Kang B., Gao G., Li C., Zhang Z. (2017). GEPIA: A web server for cancer and normal gene expression profiling and interactive analyses. Nucleic Acids Res..

