# Supplementary material for: IL-7 Deficiency Exacerbates Atopic Dermatitis in NC/Nga Mice
Source: Int J Mol Sci. 2023 Jun 9;24(12):9956. doi: 10.3390/ijms24129956 (PMC10298193; doi:10.3390/ijms24129956)
Supplement: Supplementary file 1 [file ijms-24-09956-s001.zip › ijms-2443458-supplementary.pdf]

Supplementary Figures S1 and 2

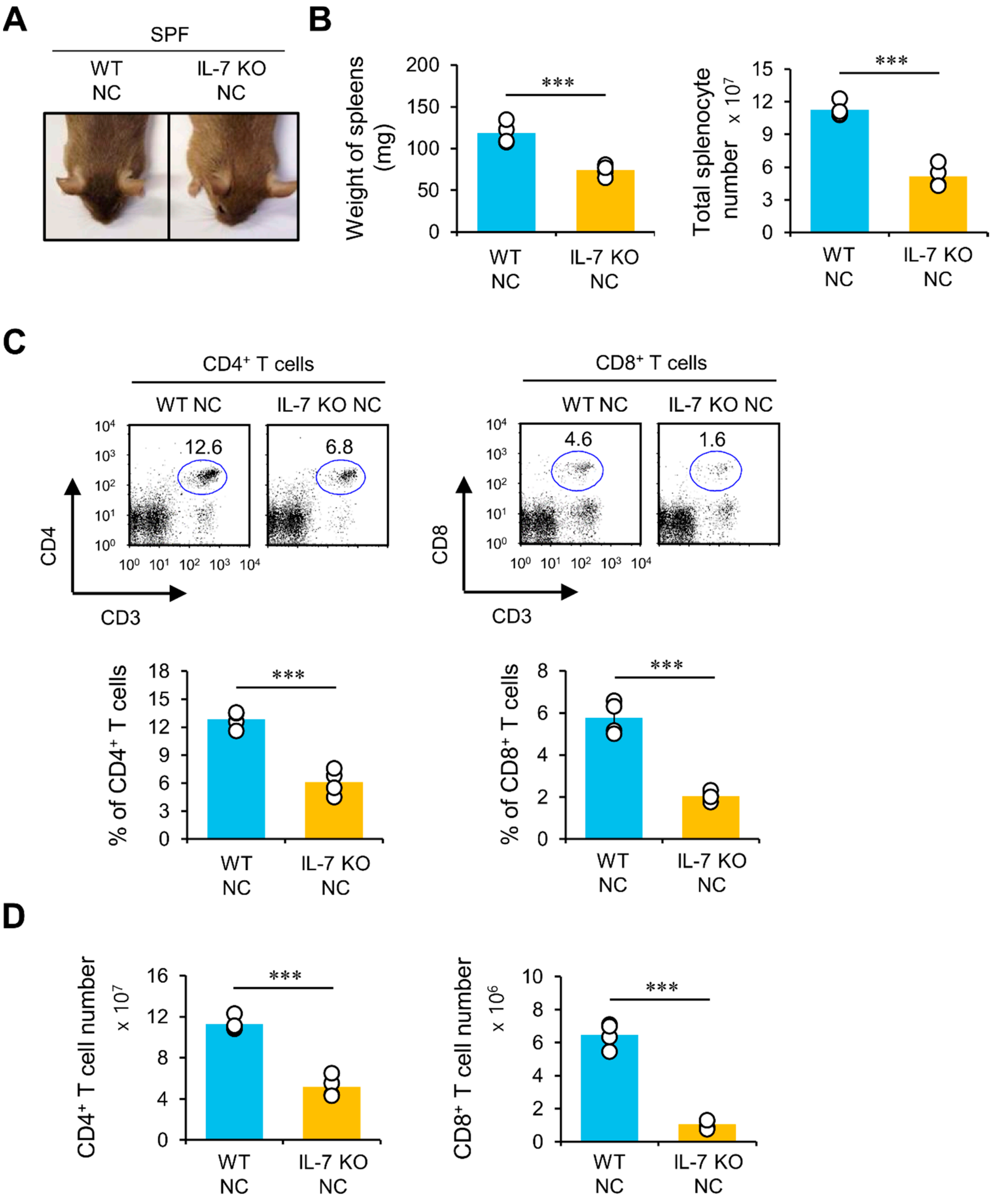

Figure S1. IL-7 Deficiency-mediated impairment of T cell development is independent of AD development

(A-D) The clinical symptoms of WT and IL-7 KO NC mice were measured to monitor the onset of AD at six weeks of age under SPF conditions. (A) Representative skin

lesions. (B) Spleen weight and splenocyte number of these mice. The percentages (C) and the absolute total cell numbers (D) of both $CD4^+$ and $CD8^+$ T cells were analyzed via flow cytometry. The mean values ± SD (n = 4 in A-D; per group in the experiment; Student's t-test; *** $p < 0.001$) are shown. One representative experiment of two experiments is shown.

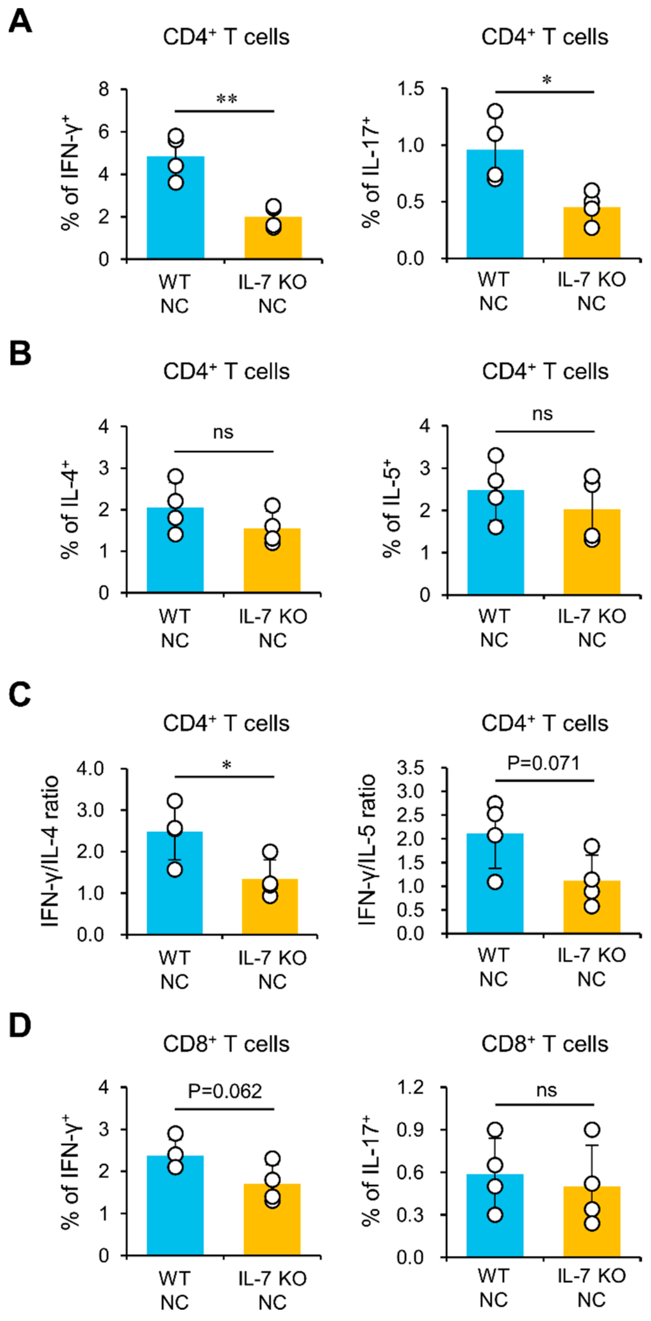

Figure S2. The effect of IL-7 deficiency on CD4 T cell differentiation in NC mice under SPF conditions

(A-D) Splenocytes were prepared from WT and IL-7 KO NC mice at six weeks of age. (A) IFN-γ- and IL-17-producing subpopulations among splenic CD4$^+$ T cells from each group were determined by flow cytometry. (B) IL-4- and IL-5-producing subsets among splenic CD4$^+$ T cells were evaluated by flow cytometry. (C) The ratio of the IL-

4- or IL-5-producing population to the IFN-γ-producing population in splenic $CD4^+$ T cells was evaluated by flow cytometry. (D) IFN-γ- and IL-17-producing subsets among splenic $CD8^+$ T cells were evaluated by flow cytometry. The mean values ± SD ($n = 4$ in A-D; per group in the experiment; Student's *t*-test; * $p < 0.05$, ** $p < 0.01$) are shown. One representative experiment of two experiments is shown. ns, not significant.
